# Microstructure, Texture and Mechanical Properties of AZ31 Magnesium Alloy Fabricated by High Strain Rate Biaxial Forging

**DOI:** 10.3390/ma13143050

**Published:** 2020-07-08

**Authors:** Yuanzhi Wu, Jizhao Liu, Bin Deng, Tuo Ye, Qingfen Li, Xiaotao Zhou, Hongji Zhang

**Affiliations:** 1 Research Institute of Automobile Parts Technology, Hunan Institute of Technology, Hengyang 421002, China; 2013001767@hnit.edu.cn (Y.W.); 1986001102@hnit.edu.cn (J.L.); 2005001476@hnit.edu.cn (B.D.); 2002000165@hnit.edu.cn (X.Z.); zhang_hongji@163.com (H.Z.); 2 School of Mechanical Engineering, Hunan Institute of Technology, Hengyang 421002, China

**Keywords:** AZ31 alloy, high strain rate biaxial forging, twinning, twining induced dynamic recrystallization, texture, mechanical property

## Abstract

High strain rate biaxial forging (HSRBF) was performed on AZ31 magnesium alloy to an accumulated strain of ΣΔε = 1.32, the related microstructure, texture and mechanical properties were investigated. It was found that the microstructure evolution can be divided into two steps during HSRBF. In the early forging processes, the refinement of the grain is obvious, the size of ~10 μm can be achieved; this can be attributed to the unique mechanisms including the formation of high density twins ({10$\bar{1}$2} extension twin and {10$\bar{1}$1}-{10$\bar{1}$2} secondary twin) and subsequently twining induced DRX (dynamic recrystallization). The thermal activated temperature increases with the increase of accumulated strain and results in the grain growth. Rolling texture is the main texture in the high strain rate biaxial forged (HSRBFed) alloys, the intensity of which decreases with the accumulated strain. Moreover, the basal pole rotates towards the direction of forging direction (FD) after each forging pass, and a basal texture with basal pole inclining at 15–20° from the rolling direction (RD) is formed in the full recrystallized HSRBFed alloys. The grain refinement and tiled texture are attributed to the excellent strength and ductility of HSRMBFed alloys with full recrystallized structure. As the accumulated strain is ΣΔε = 0.88, the HSRMBFed alloy displays an outstanding combination of mechanical properties, the ultimate tensile strength (UTS) is 331.2 MPa and the elongation is 25.1%.

## 1. Introduction

Magnesium alloys are potential materials in airplanes and automobiles, they have good machinability, excellent damping capacity and favorable recycling capability, besides which, the advantage of light weight can save energy and reduce emissions [1,2]. However, magnesium alloy is a kind of hexagonal close packed (hcp) lattice metal, and the limited slip systems leads to the poor performance of formability. Accordingly, low strain rate is adopted for magnesium alloys during the deformation, and as a result, it is costly and inefficient to produce magnesium alloy applications with traditional deformation processes [3]. In recent research, high strain rate deformation was successfully carried out on Mg-Al-Zn [4,5], Mg-Zn-Zr [6,7], Mg-RE [8,9] alloys and pure magnesium [10]. These research studies reported that high strain rate facilitates high density twinning which subsequently induces dynamic recrystallization (DRX); the twinning and DRX can release stress and consume strain energy induced by plastic deformation, and consequently results in remarkable improvement in the formability of the alloys. Based on the innovations, high strain rates rolling or forging were developed to produce wrought magnesium. Owing to the high economic impact of forging in produce bulk material for industrial applications, a series of works related to high strain rate forging of magnesium alloys have been done in recent years. Li et al. [11] produced an AZ31 alloy by rapid uniaxial forging and studied the microstructure evolution; they found that the formation of complex twins is a main reason for the decline of stored energy for grain refinement. Chen et al. produced an AZ61 [12,13] and an Mg-Gd-Y-Zr [14] alloy by adopting small strain impact multidirectional forging; the related microstructure, texture and mechanical properties of the forged alloys have been studied. In our previous studies, we have successfully produced ZK60 [15], ZK21 [16] and AZ31 [17] magnesium alloys through high strain rate triaixal forging, and the results prove that those alloys display excellent balance of strength and ductility resulting from grain refinement.

It is well known that, different strain paths during forging lead to different metal plastic flows, which consequently results in different shapes and properties [18]. For example, the metal flows to the radius and axial direction in uniaxial and biaxial forging, respectively, while no obvious dimension change takes place in triaxial forging because of the cycle change of the three orthogonal forging directions. To date, the microstructure and properties of magnesium alloys fabricated by high strain rate uniaxial forging [11] and triaxial forging [12,13,14,15,16,17] have been investigated. High strain rate biaxial forging (HSRBF) is a kind of the severe plastic deformation (SPD) techniques; however, the microstructure, texture and properties of magnesium alloy fabricated by this method are still unknown. In this work, HSRBF was performed on AZ31 magnesium alloy, and the related microstructure, texture and mechanical properties were investigated.

## 2. Experimental Procedures

**Figure 1:**
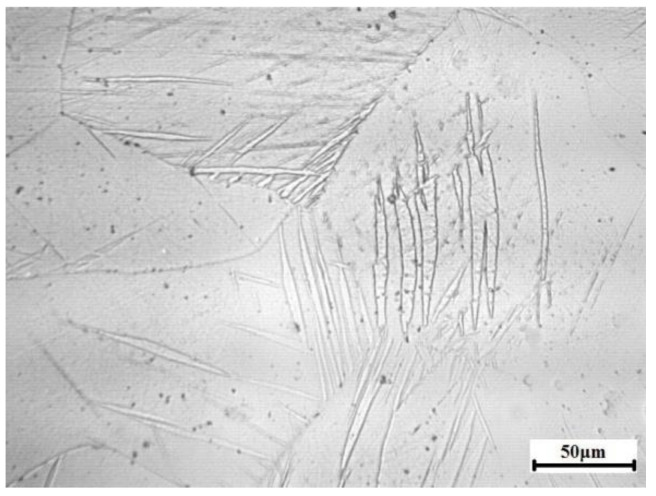
Optical microstructure characteristic of homogenized AZ31 magnesium alloy.

**Figure 2:**
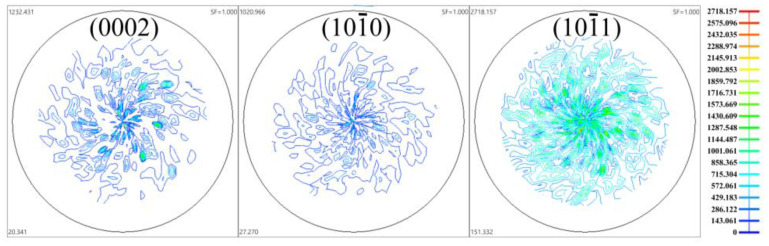
(0002), (10$\bar{1}$0) and (10$\bar{1}$1) pole figures of homogenized AZ31 magnesium alloy.

A commercial AZ31 magnesium alloy with the chemical composition of Mg-3%A1-1%Zn-0.3%Mn was selected in the present study. The as-cast billets were homogenized at 400 °C for 12 h followed by water quenching. The homogenized material was characteristic of coarse grains with grain size of ~400 μm, and few twins were detected (Figure 1). X-ray diffraction results show that the homogenized material has a nearly random texture (Figure 2), the relative intensities of the (0002), (10$\bar{1}$0), and (10$\bar{1}$1) peaks (*I*_(0002)_:*I*_(10$\bar{1}$0)_:*I*_(10$\bar{1}$1)_ = 1:0.83:2.21) were comparable to those of a completely random Mg powder (*I*_(0002)_:*I*_(10$\bar{1}$0)_:*I*_(10$\bar{1}$1)_ = 1:0.85:2.44).

**Figure 3:**
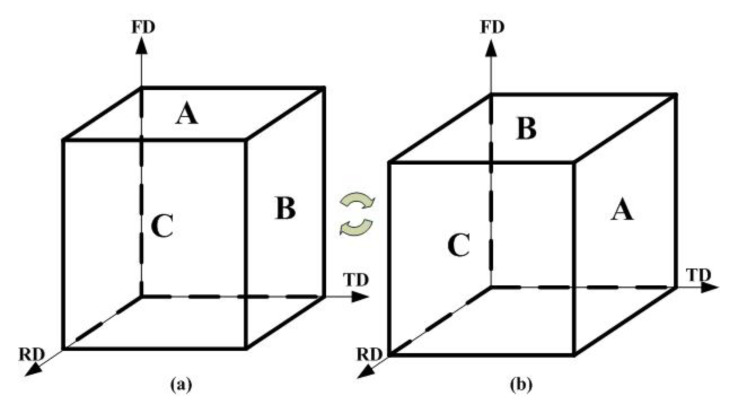
Schematic drawings of the high strain rate biaxial forging (HSRBF) process (**a**) odd number passes (**b**) even number passes.

Rectangular samples for HSRBF were machined from the homogenized ingot, and the height, width and length for the sample was 40, 35 and 35 mm, respectively. The samples were heated in a muffle furnace before forging, and the hold temperature was 350 °C for 5 min. An air hammer was used to conduct biaxial forging along two orthogonal directions in turn as illustrated in Figure 3, and the initial forging direction was parallel to the height direction of the sample. The forging speed of the hammer was 5 m/s, and the forging strain rate was calculated as about 100/s. The samples were forged to different heights (h) with different reductions (λ) in each passes, and the pass strain was calculated by the following Equation (1):

(1) $$∆\varepsilon =\ln\frac{h+\lambda }{h}$$

A pass strain of Δε = 0.22 was obtained by accurate controlling of the pass reduction (*λ*), and HSRBF was carried out to an accumulated strain of ΣΔε = 1.32, i.e., 6 passes of forging. The billets stretched along the rolling direction (RD) after HSRBF, and no obvious crack was observed.

The central section of the specimens that was perpendicular to the forging direction (FD) was selected to make different microstructure characterizations. Optic microstructures were observed using an optical microscope (OM, AX10, Zeiss, Hengyang, China) after etched with a solution of 1 g oxalic acid, 1 mL nitric acid and 98 mL water. Electron back-scatter diffraction (EBSD) observation was conducted on a scanning electron microscope (SEM, EVO18, Zeiss, Hengyang, China) at 20 kV, and 1.5 mm was selected as a step size in the measure of the orientation imaging. The Schultz reflection method was carried out on X-ray diffraction (Smartlab, Rigaku, Hengyang, China) to analysis texture. Dog-bone like tensile specimens were machined with a gauge length of 10 mm, and abrasive papers were utilized to polish the surfaces of tensile specimens. A tensile test was conducted under a constant tensile rate of 0.5 mm/min at room temperature, and the tensile direction was parallel to RD. In order to make sure of the repeatability, three experiments for each condition were conducted. The fracture characters of the tensile samples were observed on SEM.

## 3. Results and Discussion

### 3.1. Microstructure Evolution

**Figure 4:**
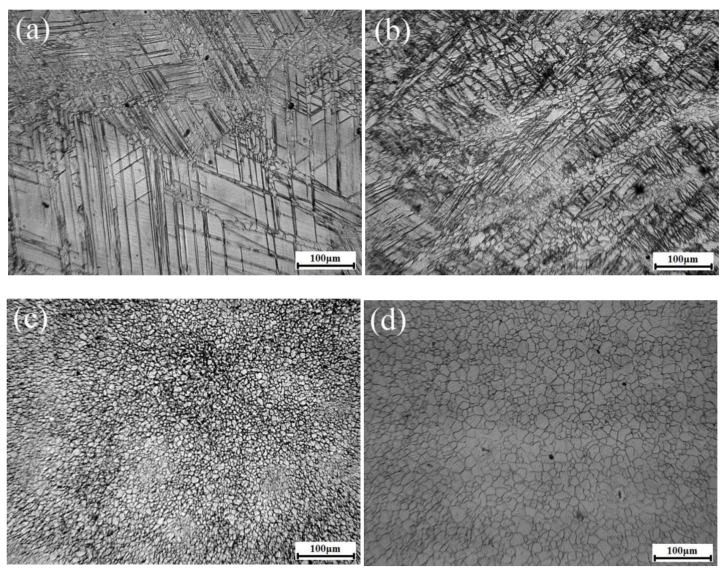
Microstructure of HSRBFed AZ31 alloys with different accumulated strains (**a**) 0.22, (**b**) 0.44, (**c**) 0.88, (**d**) 1.32

Shown in Figure 4 is the microstructure evolution of the AZ31 alloy during HSRBF. It is observed that the twins were extensively developed at the core of the initial grain and divided the initial coarse grains into finer twin platelets. Meanwhile, the development of dynamic recrystallization (DRX) is found at the twins and grain boundaries, and a few amounts of DRX grains can be observed as the accumulated strain of ΣΔε = 0.22 (Figure 4a). Both the DRX fraction and twin density increase with the accumulated strain. As the accumulated strain obtains ΣΔε = 0.44, finer twin platelets and more DRX grains were detected as shown in Figure 4b. With the further increasing of the accumulated strain, the twin plates were replaced by DRX due to its extensive development. As the accumulated strain of ΣΔε = 0.88, a fine DRX structure with the average grain size less than 10 μm was found in Figure 4c. With the further increase of accumulated strain the DRX grains grew obviously. As shown in Figure 4d, when the accumulated strain is 1.32, some of the grains were found to be more than 20 μm.

**Figure 5:**
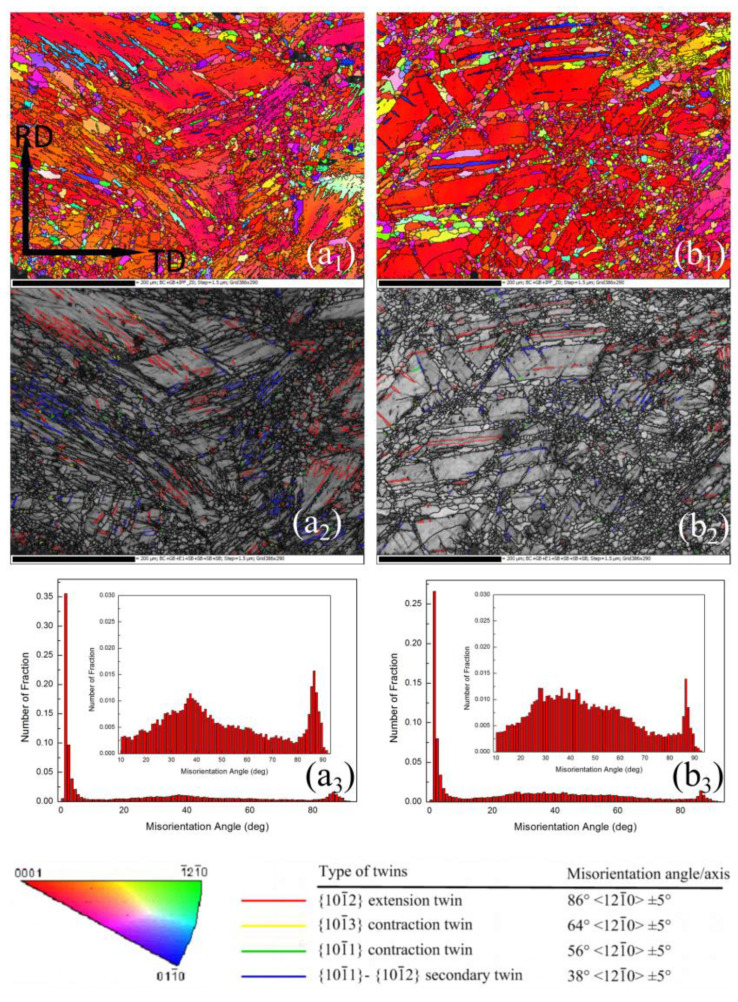
Electron back-scatter diffraction (EBSD) results of AZ31 alloys HSRBFed to different accumulated strains (**a**) 0.22, (**b**) 0.44, including inverse pole figure maps (**a_1_**,**b_1_**), boundary misorientation maps (**a_2_**,**b_2_**) and misorientation angle distributions (**a_3_**,**b_3_**).

Shown in Figure 5 are the EBSD inverse pole figure (IPF) maps, boundary misorientation maps and misorientation distribution maps of AZ31 alloys HSRBFed to accumulated strains of ΣΔε = 0.22 and 0.44. It can be seen that the microstructure detected by EBSD (Figure 5a_1_,a_2_,b_1_,b_2_) was similar to that observed by OM, in the twinned regions and at grain boundaries some DRX grains were detected, and the fraction of DRX increased significantly with the increase of accumulated strain. Moreover there are two peaks around 38°and 86° in the misorientation distribution map (Figure 5a_3_,b_3_), indicating that {10$\bar{1}$2} and {10$\bar{1}$1}-{10$\bar{1}$2} twins develop extensively in the alloy, and therefore both {10$\bar{1}$2} and {10$\bar{1}$1}-{10$\bar{1}$2} twins are the predominant twin at the early stage of HSRBF. Zhu et al. [19] and Li et al. [11] have reported the same results about magnesium alloys produced at a high strain rate. It is clear that deformation twins are formed with high density at high strain rates forging; meanwhile, the boundary number of twins is higher than that of the grain. Therefore, the DRX within twins plays a key role during HSRBF. The twinning induced DRX (TDRX) can be defined as the DRX mechanism within twins, and the twin boundaries can impede the motions of dislocation and provide the driving force for DRX, which can lead to a great grain refinement in magnesium alloys. Therefore, the formation of high density twins, and subsequently TDRX, results in the grain refinement of the studied AZ31 alloy.

**Figure 6:**
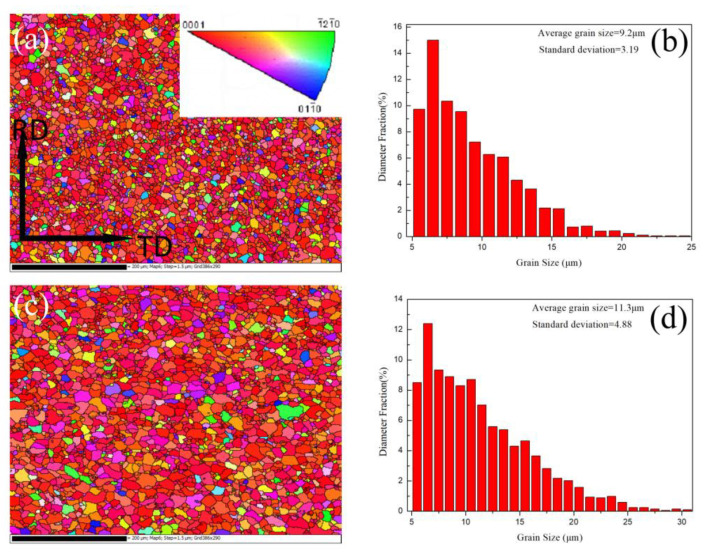
Results of AZ31 alloys HSRBFed to different accumulated strains (**a**) and (**b**) 0.88, (**c**) and (**d**) 1.32, including inverse pole figure maps and grain size distributions.

The IPF maps and grain size distribution maps of AZ31 alloys HSRBFed with accumulated strain of ΣΔε = 0.88 and 1.32 are shown in Figure 6. At the accumulated strain of ΣΔε = 0.88 a full recrystallized structure was detected, the average grain size was 9.2 μm with a standard deviation of 3.19 μm, no twin was found and the frequency of the small DRX grains (<10 μm) was about 65% while the frequency of the large DRX grains (>15 μm) was about 7%. Meanwhile, as the ΣΔε = 1.32 the corresponding average grain size was 11.4 μm, the related standard deviation was 4.88 μm and the frequency of the small DRX grains (<10 μm) was about 47% while the frequency of the large DRX grains (>15 μm) was about 22%. It was observed that in the full recrystallized structure nearly half of the DRX grains were smaller than 10 μm, and the frequency of small DRX grains decreased, as the increase of accumulated strain and the frequency of large DRX grains increased significantly.

As mentioned above, the microstructure evolution of the AZ31 alloy during HSRBF can lead to the refinement and growth of the grain, which was different from alloys forged at low strain rates as reported in references [20,21,22,23]. Firstly, much higher critical strains that control the degree of homogeneous DRX structure are found in references [20,21,22,23]. It is found that the critical equivalent strains to achieve a full recrystallized structure are 4.5, 2.4 and 3.4 for WE43 [20,21], AZ80 [22] and AZ61 [23] alloys, respectively; however, in the present study the equivalent strain is only 0.88. The same finding was reported by Li et al. [11] in the rapid forging process of AZ31 magnesium alloy. During high strain rate deformation, the unique deformation mechanisms can result in the lower equivalent strain, which includes the formation of high density twins and subsequently TDRX. It has been theoretically and experimentally proved that twinning is the dominant mechanism that is responsible for plastic deformation at high temperature deformation, because of the limited slip system in hexagonal closed-packed (HCP) magnesium alloys [24]. During deformation, the twinning and dislocation slip is in a competitive relationship, and the time for dislocation to slip is limited, therefore, twinning is more extensive at high strain rate deformation [7]. Moreover, it is reported that twins frequently form with effective interface velocity, which are appreciable fractions of the velocity of sound, and it is reasonable to infer that the twins can form rapidly [25]. As a result, twins formed with a high rate and divided the original coarse grain into finer twin plates. Besides, the {10$\bar{1}$2} twin and {10$\bar{1}$1}-{10$\bar{1}$2} double twin together with stacking faults formed at high strain rate forging can facilitate the formation of low-angle grain boundaries, which can subsequently transit into high-angle grain boundaries, and form DRX grains at twins at low strain [11,26]. As shown in Figure 4c and Figure 6a,b at the equivalent strain of 0.88, the previous twins were replaced by full recrystallized structures rapidly. Secondly, with further deformation, the grain size of the alloys which are forged at low strain rate undergo a slight change due to the completion of DRX [20,21,22,23], while the grains grew obviously with the deformation pass after a full recrystallized structure was obtained in the present research. The temperature increment caused by the adiabatic heating in high strain rate deformation may aid the grain growth. During high strain rate deformation, the energy from plastic deformation and friction convert into heat, while there is almost no heat loss due to the short deformation time, and thus an adiabatic heating condition is obtained which may lead to DRX grain growth [27].

### 3.2. Texture

**Figure 7:**
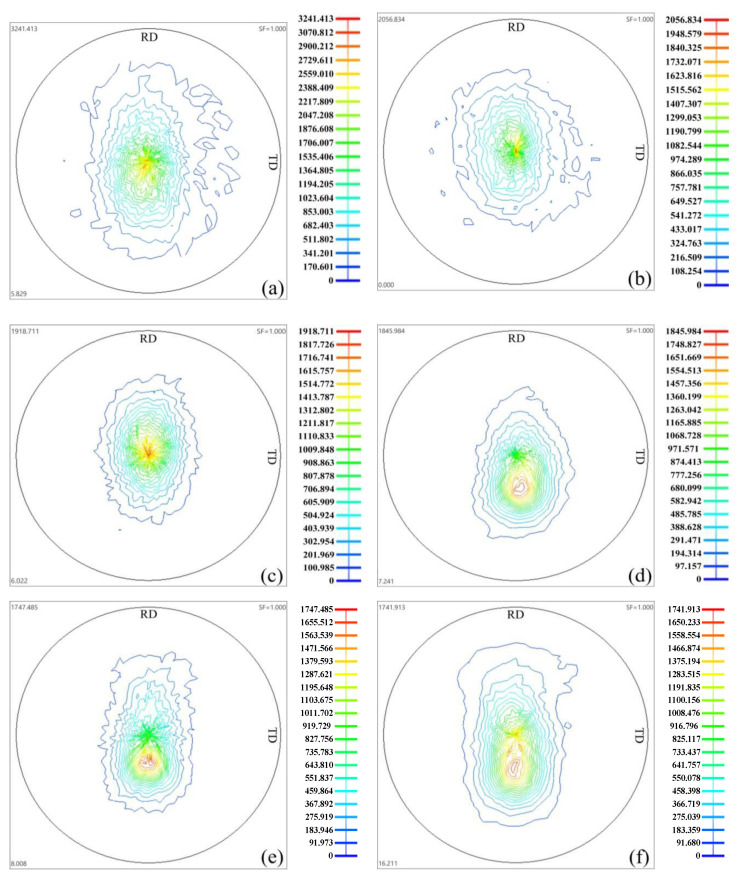
(0002) pole figure of AZ31 alloys HSRBFed to different accumulated strains (**a**) 0.22, (**b**) 0.44, (**c**) 0.66, (**d**) 0.88, (**e**) 1.1, (**f**) 1.32.

Figure 7 displays the (0002) pole figures of the AZ31 alloy HSRBFed with different accumulated strains. As can be seen in Figure 7, some important trends were obtained. Firstly, the HSRBFed alloy shows a characteristic of typical hot-rolled texture, i.e., a strong basal texture with a circle-shaped distribution of {0002} orientation. Secondly, the basal pole rotates towards the FD during each pass, and in detail, at the early forging pass, the basal pole is parallel to FD; however, as the accumulated strain exceeds 0.88, it inclines at about 15–20° from the FD towards the RD. Thirdly, the intensity of the basal rolling texture undergoes great changes, and the intensity of the basal texture decreases with the increase of accumulated strain. Furthermore, a spread in the basal pole towards the direction of the RD is apparent as the accumulated strain is higher than 0.66.

During uniaxial compression, magnesium tends to form a basal texture with the c-axis parallel to the loading direction, and the (0002) pole rotates towards the compression direction owing to the development of the {10$\bar{1}$2} twin even in small compression strains [28]. It is observed from Figure 5 that {10$\bar{1}$2} twin developed obviously at the early forging stage; as a consequence, a typical rolling texture formed as shown in Figure 7a,b. The red color in the inverse pole figures (Figure 5a_1_,b_1_) also confirms the strong basal texture as the alloys HSRBFed to the accumulated strain of ΣΔε = 0.22 and 0.44. With further increase of accumulated strain, DRX developed fast at twins and the fraction of the DRX grain increased. It was reported that during the hot deformation of magnesium alloys TDRX was of great importance in texture weakening [29]; therefore, the intensity of the texture decreased with the accumulated strain. Nie et al. [30] have revealed similar results in the process of forming the AZ91 alloy via multidirectional forging. Moreover, as the vast majority of grains are smaller in size, the non-basal slips can be activated, and consequently lead to the spread of the basal pole [31]. Therefore, as the accumulated strain went beyond 0.66, a basal spread towards direction of the RD was detected.

It is acknowledged that a magnesium alloy with tiled texture displays excellent formability; therefore, basal pole inclining is widely used in tailing the texture of a magnesium alloy. Shear deformation is one of the effective methods to achieve tilted-basal texture, which is commonly seen in processes such as equal channel angular pressing (ECAP), differential speed rolling (DSR) and friction stir processing (FSP), and it is reported that the inclinations were 20–45°, 5–15° and up to 55° in ECAP, DSR and FSP alloys, respectively [32]. It was interesting to find that the basal pole inclined at about 15–20° away from the direction of FD after HSRBF, i.e., it is feasible to process a magnesium alloy with a weakened texture by HSRBF. The following reasons can ascribe to the tiled texture [33]. Firstly, the slip in different sample planes is activated for a pass instead of being restricted to a single plane. Secondly, the alteration of forging directions leads to the activation of different strain paths. Thirdly, the DRX that has been discussed above belongs to reorientation during HSRBF.

### 3.3. Mechanical Properties

**Figure 8:**
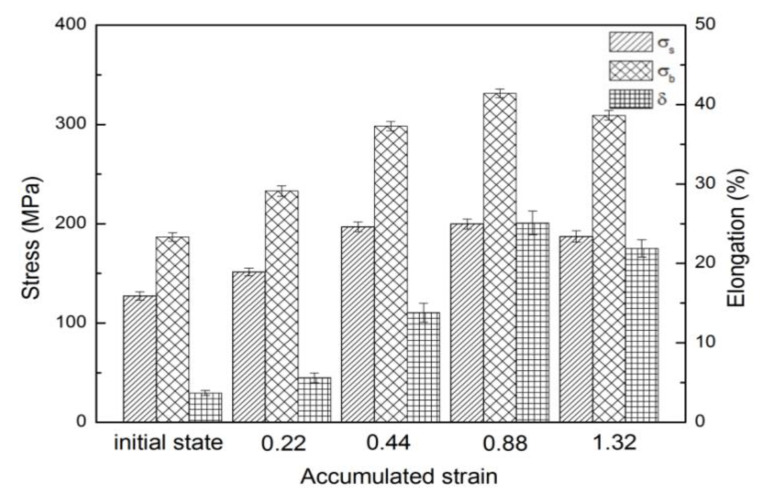
Mechanical properties of AZ31 alloys HSRBFed to different accumulated strains.

Figure 8 shows the mechanical properties of HSRBFed samples, and the corresponding tensile data is listed in Table 1, namely, yield strength (YS) σ_s_, ultimate tensile strength (UTS) σ_b_ and elongation δ. The alloy in its initial state exhibits poor mechanical properties. However, it is obvious from Figure 8 and Table 1 that the strength and elongation increase with the strain as the accumulated strain is lower than 0.88, but decrease slightly with further forging. An excellent combination of mechanical property with UTS of 331.2 MPa and elongation of 25.1% was achieved at ΣΔε = 0.88. Take the microstructure and texture discussed above in consideration: the variation of strength and ductility during HSRBF may be caused by the grain refinement and the texture weakening. The DRX volume fraction increases with the increase of the accumulated strain at the early forging pass, and the motion of dislocation would be blocked by the high density of grain boundaries; as a result, the strength rises apparently. On the other hand, the grain refinement together with texture weakening can result in the increase of plastic coordinate capability, which can be made attributable to the improvement of ductility at the early forging process. It is reported that the formation of inclined basal texture can contribute to the increased ductility of magnesium alloys by promoting basal slip [34]. On the contrary, the average grain size of the HSRBFed alloy grew from 9.2 μm to 11.4 μm with further continuous forging, which indicated a weakened dislocation blocking and less plastic coordination, which finally resulted in the decline of strength and ductility. It is of great value to note that HSRBF can achieve grain refinement and basal pole inclining, and the alloys with a full recrystallized structure display outstanding strength and elongation. It is acknowledged that multiaxial forging is the easiest severe plastic deformation (SPD) technique since it does not require any special device and has the potential to process materials on a large scale [35]. Therefore, HSRBF was an efficient way to product bulk magnesium alloys with excellent strength and ductility in comparison to other forming techniques such as equal channel angular pressing (ECAP), high pressure torsion (HPT) and cyclic extrusion and compression (CEC).

**Figure 9:**
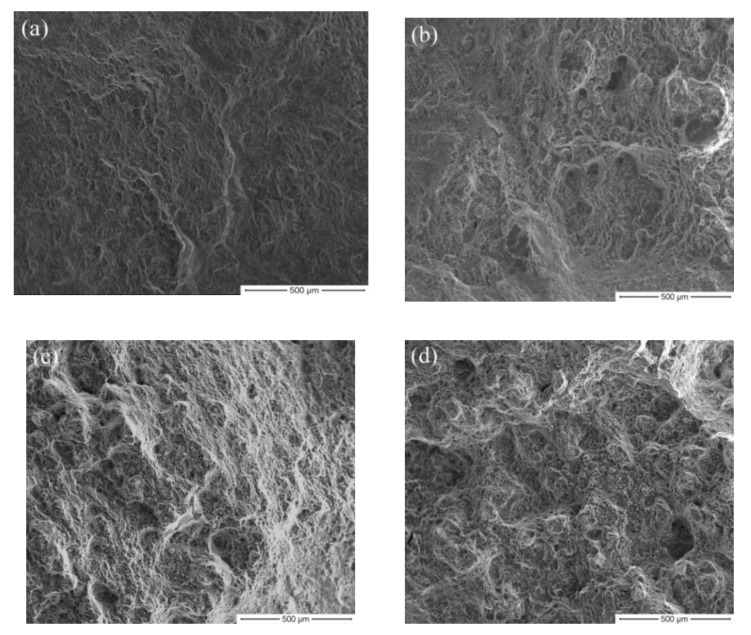
Images of tensile fracture for samples HSRBFed to different accumulated strains (**a**) 0.22, (**b**) 0.44, (**c**) 0.88, (**d**) 1.32.

Figure 9 shows tensile fracture images for alloys HSRBFed to different accumulated strains. As shown in Figure 9a, typical quasi-cleavage was observed in the alloy HSRBFed to ΣΔε = 0.22. A number of tearing ridges and cleavage steps were found in the local position of the fracture surface, but the dimples were found with low quantity. As the accumulated strain increases, as illustrated in Figure 9b, the number of dimples increases; however, almost no tearing ridge on the fracture of the HSRBFed alloy can be detected, which indicates a better ductility. As shown in Figure 9c,d, with the alloys HSRBFed to an accumulated strain of ΣΔε = 0.88 and 1.32, no obvious tearing ridges can be found at the fracture surface of the full recrystallized alloys and the entire sample was covered by large amount of dimples, which is a characteristic of fractures with excellent ductility. Take the mechanical properties given in Figure 8 and Table 1 into consideration: the fracture images are in good agreement with the ductility evolution during HSRBF.

## 4. Conclusions

The microstructure, texture and mechanical properties of the AZ31 alloy during high strain rate biaxial forging (HSRBF) were investigated. The main conclusions were listed as below.

- The microstructure evolution during HSRBF can be divided into two steps, i.e., grain refinement in the early stage and grain growth with further increase of accumulated strain. A homogeneous fine DRX structure with the average grain size of 9.2 μm was obtained as accumulated strain was 0.88, implying that DRX developed at lower strain during HSRBF.
- The formation of high density twins, and subsequently twining induced DRX, leads to the grain refinement, and {10$\bar{1}$2} and {10$\bar{1}$1}-{10$\bar{1}$2} twining are the predominant twinning mechanisms in magnesium alloy forged at high strain rate.
- The main texture in the HSRBFed alloys is a typical hot-rolled texture, and the intensity of the texture decreases with increasing of the accumulated strain. Moreover, the basal pole rotates towards the direction of forging direction (FD) after each pass, and a basal texture with a basal pole inclining at 15–20° form the rolling direction (RD) formed in the full recrystallized HSRBFed alloy.
- The strength and elongation increase with the strain as the accumulated strain is lower than 0.88, but decrease slightly with further forging, and an excellent combination of mechanical property with UTS of 331.2 MPa and elongation of 25.1% was achieved at ΣΔε = 0.88, which resulted from the combined effects of grain refinement and weakened basal texture. Therefore, HSRMF is an efficient way to produce strong and ductile wrought AZ31 alloy.

## Figures and Tables

**Table 1:** Mechanical properties of AZ31 alloy HSRBFed to different accumulated strains.

Accumulated Strain	σ_s_ (MPa)	σ_b_ (MPa)	δ (%)
initial state	127.2 ± 4.1	186.6 ± 4.4	3.7 ± 0.3
0.22	151.3 ± 3.9	233 ± 5.2	5.6 ± 0.6
0.44	196.8 ± 4.8	298.3 ± 4.7	13.8 ± 1.2
0.88	199.7 ± 4.9	331.2 ± 4.3	25.1 ± 1.5
1.32	187.2 ± 5.8	309.1 ± 4.9	21.9 ± 1.1
